# Novel genetic variants in *miR-191 *gene and familial ovarian cancer

**DOI:** 10.1186/1471-2407-10-47

**Published:** 2010-02-18

**Authors:** Jie Shen, Richard DiCioccio, Kunle Odunsi, Shashikant B Lele, Hua Zhao

**Affiliations:** 1 Department of Cancer Prevention and Controls, Roswell Park Cancer Institute, Buffalo, NY 14263, USA; 2 Department of Cancer Genetics, Roswell Park Cancer Institute, Buffalo, NY 14263, USA; 3 Department of Gynecologic Oncology, Roswell Park Cancer Institute, Buffalo, NY 14263, USA

## Abstract

**Background:**

Half of the familial aggregation of ovarian cancer can't be explained by any known risk genes, suggesting the existence of other genetic risk factors. Some of these unknown factors may not be traditional protein encoding genes. MicroRNA (miRNA) plays a critical role in tumorigenesis, but it is still unknown if variants in miRNA genes lead to predisposition to cancer. Considering the fact that miRNA regulates a number of tumor suppressor genes (TSGs) and oncogenes, genetic variations in miRNA genes could affect the levels of expression of TSGs or oncogenes and, thereby, cancer risk.

**Methods and Results:**

To test this hypothesis in familial ovarian cancer, we screened for genetic variants in thirty selected miRNA genes, which are predicted to regulate key ovarian cancer genes and are reported to be misexpressed in ovarian tumor tissues, in eighty-three patients with familial ovarian cancer. All of the patients are non-carriers of any known BRCA1/2 or mismatch repair (MMR) gene mutations. Seven novel genetic variants were observed in four primary or precursor miRNA genes. Among them, three rare variants were found in the precursor or primary precursor of the miR-191 gene. In functional assays, the one variant located in the precursor of miR-191 resulted in conformational changes in the predicted secondary structures, and consequently altered the expression of mature miR-191. In further analysis, we found that this particular variant exists in five family members who had ovarian cancer.

**Conclusions:**

Our findings suggest that there are novel genetic variants in miRNA genes, and those certain genetic variants in miRNA genes can affect the expression of mature miRNAs and, consequently, might alter the regulation of TSGs or oncogenes. Additionally, the variant might be potentially associated with the development of familial ovarian cancer.

## Background

Epithelial carcinoma of the ovary is one of the most common gynecologic malignancies in women [1]. Approximately 70% of ovarian cancer patients are diagnosed in an advanced stage. The 5-year survival rate of women with ovarian cancer is approximately 40% and has not significantly changed over the last two decades, despite advances in treatment. It has been demonstrated that prophylactic removal of ovaries in women at high risk for developing ovarian cancer is an effective prevention strategy and is especially applicable to women who are genetically predisposed to ovarian cancer. Therefore, identification of women who are predisposed to ovarian cancer at an early age is clearly needed. Unfortunately, so far, our understanding of the genetic predisposition to ovarian cancer is limited. Family history is the strongest known risk factor for ovarian cancer. Compared to a 1.6% lifetime risk of developing ovarian cancer in the general population, women with one first-degree relative with ovarian cancer have a 5% risk and women with two first-degree relatives have a 7% risk. The most common form of familial ovarian cancer can be attributed to mutations in either of the *BRCA1/2 *genes, which account for 5-13% of ovarian cancer cases in Western countries and for half of the familial aggregation of this disease [2-5]. This suggests that other unidentified genetic events may contribute to ovarian cancer.

miRNAs are short, noncoding RNAs that have been identified in the genomes of a wide range of multicellular life forms as well as viruses. miRNAs can regulate gene expression by directly binding to the 3'UTR regions of protein-encoding genes, such as tumor suppressor genes (TSGs) or oncogenes, etc. It is known that there is a widespread misexpression of miRNAs in many cancer tissues [6,7], including ovarian cancer, but little is known regarding how inherited variability in miRNA genes may predispose towards cancer. In our previous analysis in 42 patients with familial breast cancer, we identified 7 novel genetic variants in 7 miRNA genes [8]. Among these 7 variants, an A to G change in *pre-miR-30c-1 *and a C to A change in *pri-miR-17 *were only observed in patients who are non-carriers of known *BRCA1/2 *mutations. In further functional analysis of these two variants, we found both of them could cause conformational changes in the predicted secondary structures and subsequently modify the expression of mature miRNAs. More interestingly, we observed that miR-17 could bind to the 3'UTR of *BRCA1 *mRNAs in the target *in vitro *assay, suggesting that the *BRCA1 *gene is a target gene for miR-17. In addition, several studies have demonstrated that *rs2910164 *in *pre-miR-146a *is a risk modification factor for papillary thyroid carcinoma and liver cancer as well as early age of familial breast and ovarian cancer diagnosis [9-11]. Taken together, these data suggest that variations in miRNA genes may affect miRNA function, and potentially confer cancer susceptibility through regulation of key TSGs and oncogenes.

To examine whether genetic variants in miRNA genes might be able to explain some of the unidentified genetic predisposition in familial ovarian cancer, we screened genetic variants in select miRNA genes in ovarian cancer patients who are non-carriers of any known *BRCA1/2 *or MMR gene mutations. Our hypothesis is that genetic variations in miRNA genes, if inherited in the germline, could affect the levels of expression of TSGs or oncogenes and, thereby, ovarian cancer risk.

## Methods

### Study population

This study included genomic DNA samples from lymphocytes of eighty-three non-related probands from families with inherited ovarian cancer. The samples were obtained from the Gilda Radner Familial Ovarian Cancer Registry (GRFOCR). They were identified from families with inherited ovarian cancer in which at least two first or second degree relatives had epithelial ovarian cancer diagnosed at any age. All of the samples were from Caucasian women, who were non-carriers of *BRCA1/2 *or *MLH1/MSH2 *mutations, with age at diagnosis ranging from 25 to 83 years. The study was approved by the institutional IRB board.

### miRNA selection

In this study, we focused on miRNA genes which are predicted to regulate key ovarian cancer genes (such as, *BRCA1/2*, /*2*, *KRAS*, *PTEN*, *TGFβ R2*, *CTTNB1*, *BRAF*, *HNF-1β*, *MLH1 *and *MSH2*) and are reported to be misexpressed in ovarian tumor tissues. Probability of Interaction by Target Accessibility (PITA) [12], a computational algorithm that models miRNA and mRNA binding, was used to identify the putative miRNAs which are predicted to regulate key ovarian cancer genes. In addition, we have included several miRNAs whose are reported by Viola [6] to be misexpressed in several different types of tumor tissues, including ovarian tumor. In total, thirty miRNA genes were included in this study. They were *hsa-mir-132*, *140*, *143*, *154*, *185*, *197*, *205*, *370*, *9*, *139*, *200a*, *320*, *33*, *381*, *20a*, *let7*, *106b*, *188*, *198*, *216*, *191*, *221*, *222*, *26b*, *29a*, *29b*, *29c*, *93*, *19a *and *21*.

### Sequence Analysis

For each miRNA gene, we obtained its precursor sequence from the microRNA registry http://microrna.sanger.ac.uk/ and its corresponding genome region by Blast analysis http://blast.ncbi.nlm.nih.gov/Blast.cgi. The genomic region corresponding to each precursor miRNA, including at least 100 bp at the 5' and 3' ends, and covering the primary precursor of miRNA was amplified. For the miRNAs located in clusters less than 1 kb apart, the entire corresponding genomic region was amplified. The PCR cycling conditions for the assay were 94°C for 5 minutes, followed by thirty-five cycles at 94°C for 30 seconds, 60°C for 30 seconds and 72°C for 30 seconds, with a final extension step at 72°C for 7 minutes. The amplified PCR products were sequenced using the dideoxynucleotide chain termination method. Both strands of the amplified PCR products were sequenced with an ABI-PRISM 3730xl Autosequencer (Aplied Biosystems, USA) in the Roswell Park Cancer Institute (RPCI) Biopolymer Core. For quality control, random duplicate samples (5%) were run for each sequence analysis.

### Prediction of miRNA secondary structure and calculation of optimal free energy

Secondary structures and the minimum optimal free energies for each pair of wild-type and variant primary precursor of miRNA sequences, obtained from sequencing, were predicted using the RNAhybrid program http://bibiserv.techfak.uni-bielefeld.de/rnahybrid/submission.html. In all cases, the folded structures with minimal free energy were depicted. The default setting was used for the program. The instructions for the program were followed.

### Computational algorithm to predict targets

Probability of Interaction by Target Accessibility (PITA) was used to identify the putative miRNA targets [12]. PITA is a parameter-free thermodynamic model, which integrates both sequence matching and target accessibility in target prediction. This program has been shown to predict validated targets more accurately than any other program. The default setting was used for the program. The instructions for the program were followed.

### miRNA cloning, expression, and detection

To study the effect that genetic variants have on the expression levels of mature **miR-191**, we inserted the DNA fragments containing primary precursors of miRNA (Pri-miRNA) with either wildtype or variant genotype into pcDNA3.3 mammalian expression vectors (Invitrogen, Carlsbad, CA) by using the pcDNA™3.3-TOPO^® ^TA Cloning^® ^Kit. Expression constructs were transfected into SKOV-3 ovarian cancer cell lines using DOTAP Liposomal Transfection reagents (Roche, Indianapolis, IN) according to the manufacturer's recommendations. After 48 hours, miRNAs were isolated using the *mir*Vana™ miRNA Isolation Kit (Applied Biosystems, CA). The expression levels of mature microRNAs were measured by Taqman based microRNA assays (Applied Biosystems, CA) with the use of the ABI StepOne system (Applied Biosystems, CA). The relative amount of each miRNA to the amount of the tRNA for initiator methionine was determined using the equation 2^-dCT^, where dC_T _= (C_TmiRNA _- C_TU6_). Each experiment was triplicated and the mean of the triplicates was used. For each experiment, a negative control (SKOV-3 cells transfected with empty vector) was added. The signal from the negative control was deducted from the analysis.

### Quantification of target gene expression

To confirm the target prediction, we applied Real-time Taqman-based RT-PCR to quantify the expression of selected predicted target genes for miR-191 in SKOV-3 cells transfected with pcDNA3.3 plasmids with or without *miR-191 *gene using similar methods for transfection and mRNA isolation as described above. The tested target genes were *MLH1 *and *FANCD2*. ABI Taqman gene expression assays (Applied Biosystems) were used for the mRNA quantification. β-actin was used as an internal control gene to normalize the data. Each experiment was triplicated and the mean of the triplicates was used.

### Statistical analysis

Student *t *test was used to compare mean expression levels for significant differences between the wildtype and variant alleles of miR-191 in miRNA expression assay. A P-value < 0.05 was considered statistically significant, and all statistical tests were two-sided. All analyses were performed using STATA software (Version 9.0, STATA Inc, College Station, TX).

## Results

**Figure 1:**
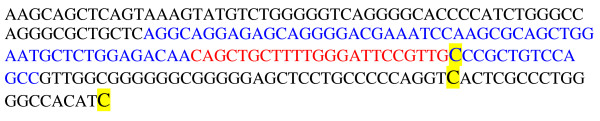
**The locations of the genetic variants in the primary precursor of *miR-191 *in relation to mature miRNA (in red) and precursor miRNA (in blue)**. The variants are highlighted in yellow.

In total, we found seven novel genetic variants in four miRNA genes (Table 1). Two variants were in the precursor of miRNA (*miR-191 *and *miR-29B-2)*, 5 were in the primary precursor of miRNA, and none were in mature miRNA, signifying the high levels of conservation. The variant allele frequencies were rare for all of the variants. Interestingly, we observed three novel variants in *miR-191 *gene, one in the precursor region and two in the primary region (Figure 1). miR-191 has been observed to be misexpressed in a variety of human tumors, including breast, ovarian, prostate, colorectal, etc. However, the functional significance of miR-191 in familial ovarian cancer is unknown. Using the PITA prediction algorithm, we found several cancer related genes are the potential targets for miR-191. They include *IGF1R, FANCC, FANCF, FANCD2*, and *MLH1*, etc.

**Table 1:** Novel Genetic variants in selected miRNA genes in ovarian cancer patients

miRNA genes	Nt Change	Location	Allele frequency	Selected target genes (examples)*
*miR -17*	C/T	Pri-miRNA	0.012	*BRCA1*, *PTEN*, *p53 *and *ATM*
*hsa-mir-191*	C/T	Pri-miRNA	0.006	*IGF1R, FANCC, FANCF, FANCD2, MLH3*
	C/G	pri-miRNA	0.012	
	C/A	Pre-miRNA	0.006	
*hsa-mir-29b-2*	C/T	pri-miRNA	0.042	*DNMT3A, IGFI, CCND2, BCL2*
	A/T	pre-miRNA	0.021	*DNMT3A, IGFI, CCND2, BCL2*
*hsa-mir-188*	T/C	pri-miRNA	0.006	*CCND2, XRCC2, IGFI, E2F*

* This non-inclusive list shows only the potential targets known to be involved in tumorigenesis. Other, unrelated, targets may exist for these miRNAs.

**Figure 2:**
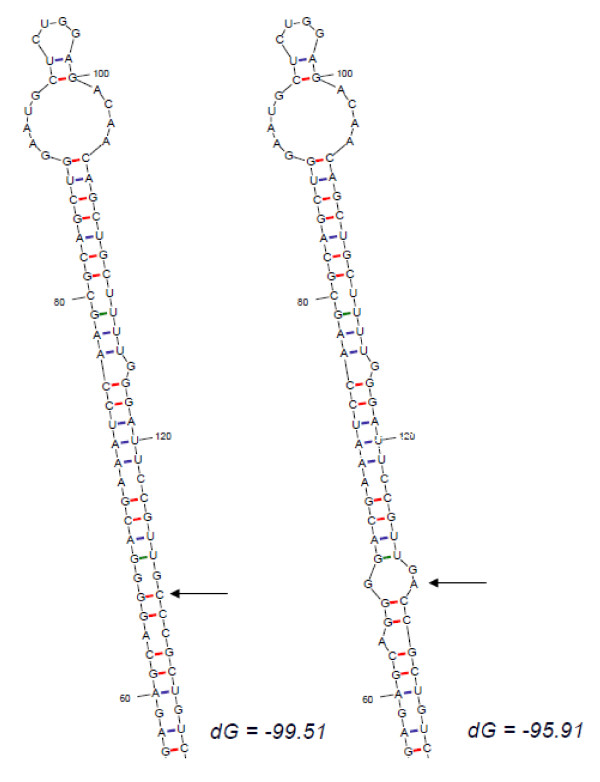
**Sequence variation in the precursor of miR-191 can translate intro structural alterations**. The RNA secondary structure was predicted by RNAHYbrid for WT pri-miR-191 and its novel variant. Only the most stable secondary structures with the lowest free energy are depicted.

Given the presence of novel genetic variants affecting the precursor of miRNA, but not the mature miRNA itself, we tested whether such variants could affect their secondary structure and thereby block processing into the functional mature miRNA. For *miR-191 *gene, the secondary structures for wildtype and the three variants were predicted. Both variants in the primary precursor region of the *miR-191 *gene didn't change the secondary structures of miR-191. On the contrary, the C to A genetic variant in the precursor of miR-191 (pre-mi191) slightly altered the secondary structure of miR-191 (Figure 2). The A allele destroyed a base-pairing, which altered the integrity of the stem and changed the secondary structure of this pre-miRNA. The optimal free energy was decreased from -49.91 Kcal/Mol for C allele to -42.1 kcal/Mol for A allele, suggesting a less stable secondary structure for A allele than C allele.

**Figure 3:**
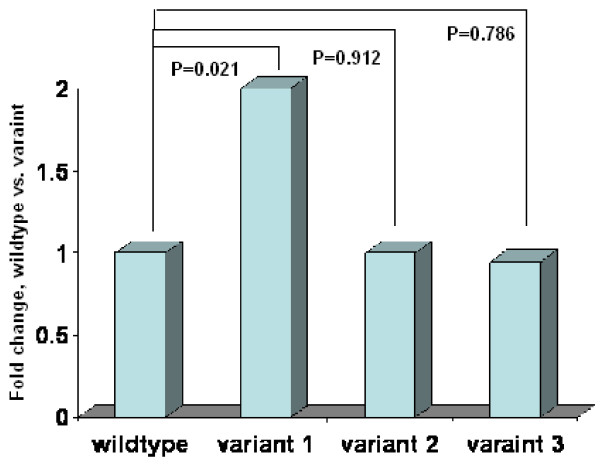
**Real-time quantitative PCR for mature miR-191 expression in the cells transfected with pcDNA3.3 vector containing miR-191 wildtype or one of three variants**. Data are means (± SD) from three independent experiments. Data are normalized with reference tRNA, as mentioned in the text. P value was calculated from a two-sided, one-sample *t *test.

Processing of miRNA precursors by the RNase Drosha requires the secondary hairpin structure characteristic of these RNA molecules and specific sequence elements within the primary precursor of miRNA. To assess the effect of the predicted changes in secondary structure on mature miRNA expression, we cloned the primary precursor of miR-191 containing C or A allele into pcDNA3.3, transfected the constructs into SKOV-3 ovarian cancer cell lines, and measured expression levels of mature miR-191 by Taqman based microRNA assays. We found that the expression levels of mature C allele miR-191 were significantly higher than those of the A allele (*P *= 0.021) (Figure 3). The expression levels of mature miR-191 were over 2 times higher in C allele than A allele, suggesting that this polymorphism in the precursor of *miR-191 *could enhance the mature miRNA production. A similar approach was used to test whether the two other genetic variant in the primary precursor of miR-191 could affect the mature miRNA expression. As expected, we didn't observe any significant changes in mature miR-191 expression between wildtype and the primary-precursor variant of *miR-191 *(Figure 3).

**Figure 4:**
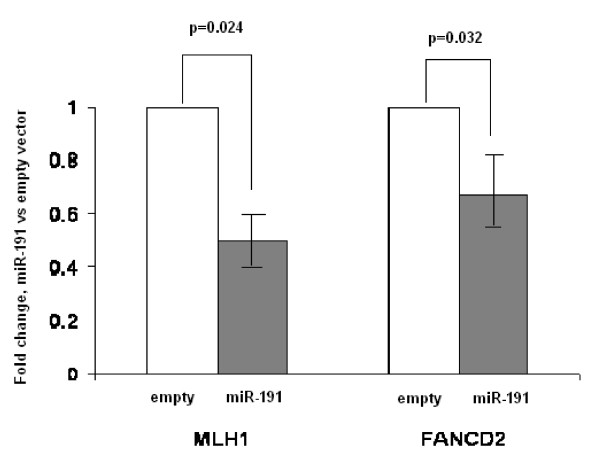
**Real time quantitative PCR for *MLH1 *and *FANCD2 *gene expression in the cells transfected with pcDNA3.3 vector containing miR-191 or the empty vector**. Data are means (± SD) from three independent experiments. Data are normalized with reference β-actin, as mentioned in the text. P value was calculated from a two-sided, one-sample *t *test.

To further confirm the target prediction, we applied Taqman-based real-time RT-PCR to detect whether the expression of MLH1 and FANCD2 were affected when SKOV-3 cells were transfected with miR-191 expression vector. Compared to SKOV-3 cells transfected with the empty vector, SKOV-3 cells transfected with miR-191 expression vector had significantly lower MLH1 and FANCD2 expression (50% for MLH1, P = 0.024; 67% for FANCD2, P = 0.032), suggesting that MLH1 and FANCD2 were target genes for miR-191 (Figure 4).

**Figure 5:**
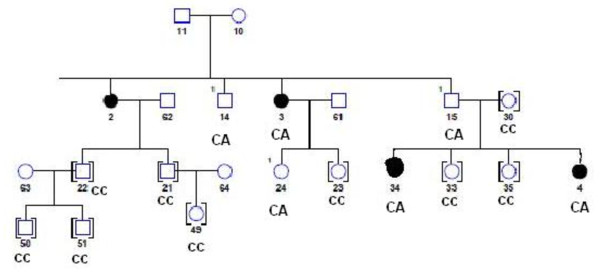
**Family pedigree structure for the proband who carries the novel variant in the precursor of *miR-191***. To simplify the structure, we only show those relatives for whom we have DNA samples. Circle: women, Square: men, and dark circle: ovarian cancer patients.

For the genetic variant in the precursor of the miR-191 gene, we further investigated the genetic inheritance of the variant in the extended family members. For the C to A change in miR-191, DNA samples for 13 relatives were available. In these 13 relatives, 5 had CA genotypes and the 8 had CC genotypes. Including the proband, three individuals who had the CA genotype were diagnosed with ovarian cancer. The relatives who had the CC genotype all appeared healthy when they were recruited. The detailed pedigree is shown in Figure 5.

## Discussion and Conclusions

Because of the particular way in which miRNA functions, by targeting a number of functionally important protein-encoding genes, it is appealing to propose that genetic variations in miRNA genes and/or their responsive elements in the target mRNAs might represent a newly described mechanism of cancer predisposition [13]. In this study, we found several novel genetic variants in the primary precursors or precursors of miRNA genes in patients with familial ovarian cancer, but who are non-carriers of any known *BRCA1/2 *or *MLH1/MSH1 *mutations, representing enriched unresolved genetic susceptibility to ovarian cancer. One rare variant in the precursor of *miR-191 *results in conformational changes in the predicted secondary structures, and consequently alter the expression of mature miRNA. In further family analysis, we found that this variant exists in four extended family members, including two individuals who were diagnosed with ovarian cancer. At the same time, family members who are not carriers of this rare variant all appeared to be cancer free when they were recruited.

Consistent with our previous study in breast cancer and other literature reports, our screening did not reveal any genetic variants in mature miRNAs, demonstrating the evolutionary conservation of miRNA genes. In order to further explore whether these rare variants exist in the general population, we genotyped 100 healthy women. As we expected, we didn't observe any of these rare variants in these healthy women. Although a "common disease-common allele" model has been accepted for most of the sporadic cancers, the situation in familial cancers might be different. So far, most of the genetic risk alleles contributing to familial ovarian cancer are rare mutations in *BRCA1/2 *or *MLH1/MSH1 *genes. No common alleles have been found to be associated with familial ovarian cancer. Therefore, just like familial breast cancer, the model that best reflects familial ovarian cancer might be a "common disease-multiple rare alleles" model. The findings from this study provide the evidence to support this model.

The contribution of rare variants with medium or low penetrance in cancer predisposition is largely unknown. On one hand, because of their rarity, their population attributive risk tends to be low. On the other hand, it has been found that rare variants are more likely to have functional significance compared to common variants. In a recent publication by the Amos group, they reported that the minor allele frequency (MAF) distribution of functional variants was shifted towards rare variants compared with the variant distribution of neutral variants [14]. They observed an inverse relationship between MAF and the proportion of functional variants, suggesting that rare variants tend to have more biological function compared to common variants. In this study, we found a rare variant in the precursor of the *miR-191 *gene that alters the secondary structure of the precursor miR-191, and thereby affect the expression of mature miR-191.

It has been hypothesized that the disturbance of the secondary structure of the miRNA precursor by sequence variations may affect the maturation process of miRNA. Consistent with this hypothesis, in this study, we found that the C to A variation causes a significant structure change in the stem region of the miR-191 precursor and results in the reduced production of mature miR-191. Similar findings have also been observed in our previous study in familial breast cancer and several other studies [8,15,16]. We found an A to G change in *pre-miR-30c-1 *and a C to A change in *pri-miR-17 *in familial breast cancer patients who are non-carriers of known *BRCA1/2 *mutations [8]. In further functional analysis of these two variants, we found both of them could cause conformational changes in the predicted secondary structures and, subsequently, modify the expression of mature miRNAs. The expression of mature miR-125a is decreased when a G:C match is replaced by a U:C mismatch in the stem region [15]. Furthermore, introduction of an artificial mutation to the stem of miR-30 and miR-21 precursors revealed that large bulges in the stem regions were detrimental to the production of miRNA [16].

Whether miR-191 plays any role in familial ovarian cancer is unknown. miR-191 is a member of the core miRNAs which are frequently misexpressed in a variety of human tumors, including, breast, ovarian, prostate, and colorectal [6]. So far, there is no report on why miR-191 is misexpressed and which target genes miR-191 might regulate. Using PITA algorithms, we found that miR-191 could potentially regulate several important genes which are related to familial ovarian cancer. They include *MLH1*, *FANCC, FANCF *and *FANCD2*. In our analysis, we found that SKOV-3 cells transfected with miR-191 expression vector had significantly lower MLH1 and FANCD2 expression (50% for MLH1 and 67% for FANCD2), compared to SKOV-3 cells transfected with empty vector. Our results provide an indirect evidence to support the target prediction. The contribution of mismatch repair (MMR) genes to familial ovarian cancer has been well-documented. A group of rare mutations in *MLH1 *gene contribute to familial ovarian cancer [17]. The relationship between the Fanconi anemia (FA) genes (*FANCC, FANCF *and *FANCD2*) and familial ovarian cancer is intriguing. It has been reported that tissue-restricted hypersensitivity to cross-linking agents is a frequent finding in ovarian cancer patients or high risk women with no *BRCA1/2 *mutations [18]. Ectopic expression of normal FANCD2 cDNA increased FANCD2 protein and induced MMC resistance in both hypersensitive cell lines tested, illustrating the key role of FANCD2 in the sensitivity of cross-linking agents. However, no FANCD2 coding region or promoter mutations were found, and there was no genomic loss or promoter methylation in FANCD2 in ovarian tumor tissues. This suggests that there might be other regulation mechanisms (such as miRNAs) which regulate FANCD2's expression in ovarian tissues.

One way to assess the genetic susceptibility of rare variants is to look at the inheritance of the variants in the families. In this study, we found that 5 out of a total of 13 relatives had CA genotypes and the other 8 had CC genotypes. Including the proband, three individuals who had the CA genotype were diagnosed with ovarian cancer. The relatives who had the CC genotype all appeared healthy when they were recruited. It appears that, to a certain degree, this rare variant in the precursor of *miR-191 *co-segregates with familial ovarian cancer, although we don't have enough families or extended family members to perform a meaningful pedigree analysis. Therefore, further screening of this rare variant in other families is necessary.

The global expression of miRNA in ovarian tumor tissues has been studied [6,7]. For example, Zhang et al. used an array comparative genomic hybridization (aCGH) approach to identify miRNA loci gained/lost in ovarian cancer [7]. Of 283 miRNA loci analyzed, 105 (37.1%) were determined to be significantly altered in their copy number in 93 primary ovarian tumor tissues. Quantitative RT-PCR demonstrated that the expression of miRNAs was consistent with the DNA copy number status for 73.1% of miRNAs, demonstrating that DNA copy number alterations may have an important effect on miRNA expression. Of interest is the loss of the *miR-17-92 *locus in not only ovarian tumor but also breast tumors and melanoma. In our study, we found a rare genetic variant in miR-17 gene. Although our study focuses on inherited genetic variants and miRNA expression studies focus on somatic alterations of miRNA expression, it would be interesting to look at the relationship between germline variants in miRNA genes and miRNA expression in ovarian tumor tissues.

There are several limitations in this study. For example, it might be interesting to look at the links between genetic variants in *miR-191 *and target gene expression in matched tumor tissues. Unfortunately, the tumor tissues are not available for these patients. It would be nice to assess the relationship between these genetic variants in miRNA genes and the patients' phenotypes, such as clinical and demographic characteristics. Unfortunately, we don't have detailed information for this study population.

This is the first report to show the existence of genetic variants in selected miRNA genes in patients with familial ovarian cancer. We found that a genetic variant in the precursor of *miR-191 *could significantly change the secondary structures and, consequently, alter the expression of mature miRNAs. Future large population or family studies are needed to assess the contribution of miRNA variants to cancer risk as well as cancer phenotypes. Advanced *in vivo *molecular assays are warranted to further confirm our findings in functional analyses. Nevertheless, our data suggest that functional variants in miRNA genes might alter the expression of mature miRNAs. It opens the possibility that miRNA genes might be novel ovarian cancer predisposition genes, although this still needs to be cautiously tested.

## Competing interests

The authors declare that they have no competing interests.

## Authors' contributions

JS carried out the molecular genetic studies and drafted the manuscript. RD, KO and SBL. SBL participated in the design of the study and manuscript preparation. HZ conceived of the study, participated in its design and coordination, performed the statistical analysis, and helped to draft the manuscript. All authors have read and approved the final manuscript.

## Pre-publication history

The pre-publication history for this paper can be accessed here:

http://www.biomedcentral.com/1471-2407/10/47/prepub

## References

[B1] Yancik R Ovarian cancer. Age contrasts in incidence, histology, disease stage at diagnosis, and mortality Cancer 1993 71 2 Suppl 517 23 8420671 10.1002/cncr.2820710205

[B2] Narod SA Ford D Devilee P Barkardottir RB Lynch HT Smith SA Ponder BAJ Weber BL Garber JE Birch JM Cornelis RS Kelsell DP Spurr NK Smyth E Haites N Sobol H Bignon Y-J Chang-Claude J Hamann U Lindblom A Borg A Piver MS Gallion HH Struewing JP Whittemore A Tonin P Goldgar DE Easton DF An evaluation of genetic heterogeneity in 145 breast-ovarian cancer families Am J Hum Genet 1995 56 254 264 7825586 PMC1801289

[B3] Ford D Easton DF Stratton M Narod S Goldgar D Devilee P Bishop DT Weber B Lenoir G Chang-Claude J Sobol H Teare MD Struewing J Arason A Scherneck S Peto J Rebbeck TR Tonin P Neuhausen S Barkardottir R Eyfjord J Lynch H Ponder BA Gayther SA Zelada-Hedman M Genetic heterogeneity and penetrance analysis of the BRCA1 and BRCA2 genes in breast cancer families Am J Hum Genet 1998 62 676 689 10.1086/301749 9497246 PMC1376944

[B4] Easton DF Bishop DT Ford D Crockfor GF Genetic linkage analysis in familial breast and ovarian cancer: results from 214 families Am J Hum Genet 1993 52 678 701 8460634 PMC1682082

[B5] Lynch HT Albano WA Lynch JF Lynch PM Campbell A Surveillance and management of patients at high genetic risk for ovarian carcinoma Obstet Gynecol 1982 59 589 596 7070730

[B6] Volinia S Calin GA Liu CG Ambs S Cimmino A Petrocca F Visone R Iorio M Roldo C Ferracin M Prueitt RL Yanaihara N Lanza G Scarpa A Vecchione A Negrini M Harris CC Croce CM A microRNA expression signature of human solid tumors define cancer gene targets Proc Natl Acad Sci USA 2006 103 2257 61 10.1073/pnas.0510565103 16461460 PMC1413718

[B7] Zhang L Volinia S Bonome T Calin GA Greshock J Yang N Liu CG Giannakakis A Alexiou P Hasegawa K Johnstone CN Megraw MS Adams S Lassus H Huang J Kaur S Liang S Sethupathy P Leminen A Simossis VA Sandaltzopoulos R Naomoto Y Katsaros D Gimotty PA DeMichele A Huang Q Bützow R Rustgi AK Weber BL Birrer MJ Hatzigeorgiou AG Croce CM Coukos G Genomic and epigenetic alterations deregulate microRNA expression in human epithelial ovarian cancer Proc Natl Acad Sci USA 2008 105 7004 9 10.1073/pnas.0801615105 18458333 PMC2383982

[B8] Shen J Ambrosone CB Zhao H Novel genetic variants in microRNA genes and familial breast cancer Int J Cancer 2009 124 1178 82 10.1002/ijc.24008 19048628

[B9] Shen J Ambrosone CB DiCioccio RA Odunsi K Lele SB Zhao H A functional polymorphism in the miR-146a gene and age of familial breast/ovarian cancer diagnosis Carcinogenesis 2008 29 1963 6 10.1093/carcin/bgn172 18660546

[B10] Xu T Zhu Y Wei QK Yuan Y Zhou F Ge YY Yang JR Su H Zhuang SM A functional polymorphism in the miR-146a gene is associated with the risk for hepatocellular carcinoma Carcinogenesis 2008 29 2126 31 10.1093/carcin/bgn195 18711148

[B11] Jazdzewski K Murray EL Franssila K Jarzab B Schoenberg DR de la Chapelle A Common SNP in pre-miR-146a decreases mature miR expression and predisposes to papillary thyroid carcinoma Proc Natl Acad Sci USA 2008 105 7269 74 10.1073/pnas.0802682105 18474871 PMC2438239

[B12] Iovino N Unnerstall U Gaul U Segal E The role of site accessibility in microRNA target recognition Nat Genet 2007 39 1278 84 10.1038/ng2135 17893677

[B13] Calin GA Croce CM MIcroRNA signatures in human cancers Nat Rev Cancer 2006 6 857 866 10.1038/nrc1997 17060945

[B14] Gorlov IP Gorlova OY Sunyaev SR Spitz MR Amos CI Shifting paradigm of association studies: value of rare single-nucleotide polymorphisms Am J Hum Genet 2008 82 100 12 10.1016/j.ajhg.2007.09.006 18179889 PMC2253956

[B15] Duan R Pak C Jin P Single nucleotide polymorphism associated with mature miR-125a alters the processing of pri-miRNA Hum Mol Genet 2007 16 1124 1131 10.1093/hmg/ddm062 17400653

[B16] Zheng Y Cullen BR Sequence requirements for microRNA processing and function in human cells RNA 2003 9 112 123 10.1261/rna.2780503 12554881 PMC1370375

[B17] Lynch HT Casey MJ Lynch J White TE Godwin AK Genetics and ovarian carcinoma Semin Oncol 1998 25 265 80 9633840

[B18] Pejovic T Yates JE Liu HY Hays LE Akkari Y Torimaru Y Keeble W Rathbun RK Rodgers WH Bale AE Ameziane N Zwaan CM Errami A Thuillier P Cappuccini F Olson SB Cain JM Bagby GC Jr Cytogenetic instability in ovarian epithelial cells from women at risk of ovarian cancer Cancer Res 2006 66 9017 25 10.1158/0008-5472.CAN-06-0222 16982743

